# Cocaine-Induced Rhabdomyolysis Causing Lumbosacral Plexopathy in an Adult Male

**DOI:** 10.7759/cureus.31613

**Published:** 2022-11-17

**Authors:** Yazeed G Sweedan, Muhammad Haroon Khilan, Ashish Jain, Rahul Rane, Saba Waseem

**Affiliations:** 1 Internal Medicine, Conemaugh Memorial Medical Center, Johnstown, USA

**Keywords:** lumbosacral plexopathy, symmetrical muscle edema, cocaine, mri, rhabdomyolysis

## Abstract

A 48-year-old male presented three days after cocaine use with acute, rapid onset of bilateral lower extremity weakness, bilateral foot numbness, acute urinary retention, and significantly elevated creatinine kinase. Further testing revealed unusual symmetrical edema with contrast enhancement on MRI of the lower extremities. The patient was diagnosed with severe non-traumatic, non-exertional rhabdomyolysis causing lumbosacral plexopathy following cocaine use. The treatment was centered around aggressive fluid resuscitation and electrolyte replacement.

## Introduction

Rhabdomyolysis is a syndrome characterized by the rapid breakdown of striated skeletal muscle, which can occur as a complication of a multitude of inciting events [1]. At first glance, its clinical presentation might seem distinctive, but with the exponential expansion of medical knowledge through research, it has become clear that rhabdomyolysis should be approached as a process with a wide spectrum of possible presentations and complications. Its presentation can range from an asymptomatic elevation in creatinine kinase to a life-threatening condition associated with an extreme elevation in creatinine kinase, electrolyte abnormalities, acute renal failure, severe muscle edema, and disseminated intravascular coagulation [1]. We present a rare case of lumbosacral plexopathy due to severe muscle edema secondary to rhabdomyolysis.

## Case presentation

A 48-year-old male with a past medical history significant for acquired hypothyroidism, compressive neuropathy of the ulnar nerve, rhabdomyolysis, and substance use disorder presented to our facility with rapid-onset, bilateral lower extremity weakness of less than 24-hour duration and three days post cocaine use. The weakness was associated with bilateral foot numbness, low back pain, urine retention, and inability to ambulate. The patient denied flu-like symptoms or diarrhea within the month preceding this hospital admission. There was no report or evidence of saddle anesthesia, urine or bowel incontinence, fever, or shortness of breath. Physical examination of the lower extremities was remarkable for grade 1/5 muscle strength and 1+ knee and ankle reflexes bilaterally. There was decreased sensation to the light touch of the feet bilaterally.

Initial laboratory testing was remarkable for high creatinine kinase, myoglobin, and creatinine. Aspartate aminotransferase, alanine aminotransferase, and lactate dehydrogenase were also found to be elevated. The patient’s thyroid-stimulating hormone was high while the free T4 was low, indicative of medication non-compliance (Table 1). Urine analysis was significant for large blood without RBCs on microscopic examination. Extensive microbiological testing was significant for past infections with Lyme disease, Epstein-Barr virus (EBV), and hepatitis C virus (HCV). The antinuclear antibody (ANA), anti-JO1 antibodies, and cryoglobulin tests were negative.

**Table 1:** Remarkable initial lab values

Lab test	Reference range and units	Patient values
Creatinine kinase	30–280 U/L	>7,800 U/L
Myoglobin	0.0–110.0 ng/mL	>20,000 ng/ml
Creatinine	0.55–1.30 mg/dL	1.5 mg/dl
Aspartate aminotransferase	5–34 U/L	312 U/L
Alanine aminotransferase	≤55 U/L	77 U/L
Lactate dehydrogenase	125–243 U/L	1,614 U/L
Thyroid-stimulating hormone	0.35–4.94 uIU/mL	41.43 ulU/ml
Free thyroxine	0.89–1.76 ng/dL	0.19 ng/dl

**Figure 1:**
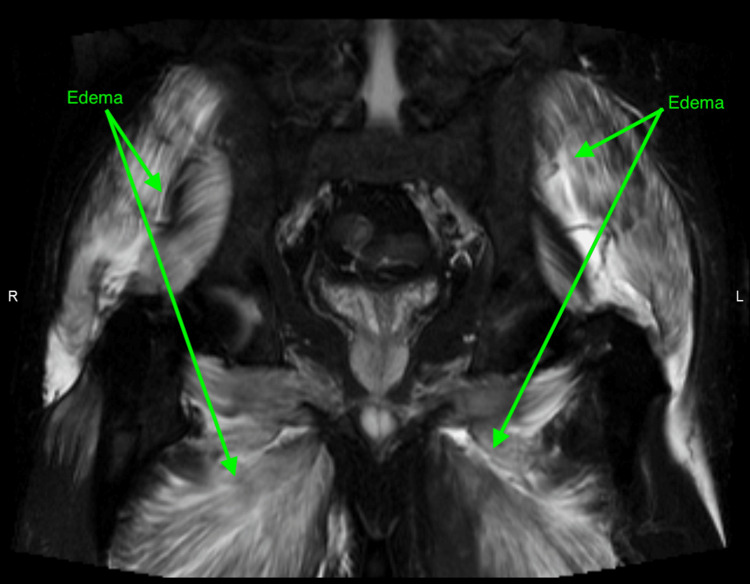
MRI of the pelvis without contrast - coronal STIR MRI: magnetic resonance imaging; STIR: short-tau inversion recovery

**Figure 2:**
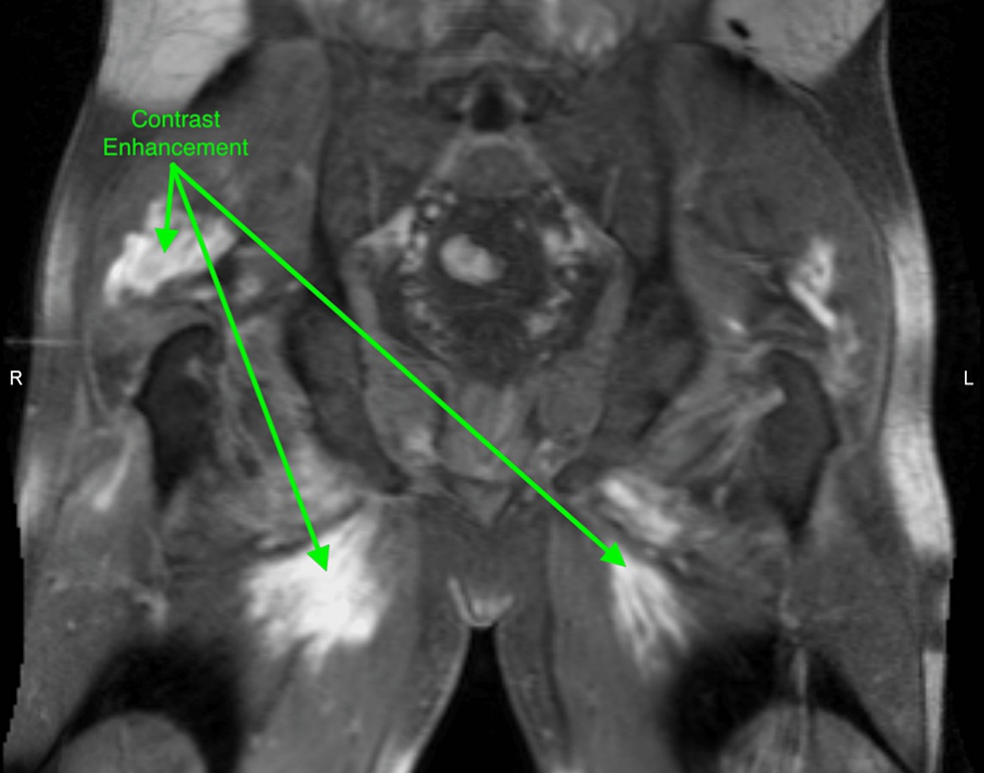
MRI of the pelvis with contrast - coronal VIBE MRI: magnetic resonance imaging; VIBE: volumetric interpolated breath-hold examination

A bedside ultrasound of the bladder after urination showed an abnormally distended bladder. Subsequently, a Foley catheter was inserted, which drained 1,500 cc of dark-colored urine. MRI of the lumbar spine with contrast was performed and showed a patent central canal and neuronal foramen with no focal disc herniation. However, it also detected abnormal posterior para-spinal, gluteal, and proximal thigh muscle signals, and enhancement without subcutaneous edema. Follow-up MRI of the pelvis and lower extremities with contrast was significant for an unusual symmetrical pattern of muscle edema with areas of contrast enhancement (Figures 1, 2). Lumbar puncture was performed with CSF studies showing a WBC count of 1/cumm and elevated protein at 100 mg/dl.

The patient’s levothyroxine supplementation was restarted. He was aggressively hydrated with IV fluids, leading to a gradual decline in serum creatinine kinase and myoglobin. On the fifth day of admission, the patient showed significant clinical improvement in lower extremity motor strength and was able to lift both legs against gravity. A successful voiding trial was performed on the same day after which the Foley catheter was removed. The patient was able to walk with assistance on the sixth day of admission. Serum creatinine kinase levels dropped below 5,000 U/L on the ninth day of admission, and the patient was subsequently discharged to a rehabilitation facility.

## Discussion

Rhabdomyolysis is a syndrome characterized by the rapid breakdown of striated skeletal muscle and the subsequent release of intracellular muscle constituents into the circulation [1]. Its triggers can be classified as traumatic, exertional, or non-traumatic, and non-exertional [1]. Patients commonly present with complaints of acute-onset myopathic symptoms including myalgia and muscle weakness, frequently associated with dark-colored urine [1]. Laboratory testing shows marked elevations of muscle enzymes alongside myoglobinuria on urine analysis [1]. Acute kidney injury (AKI) may develop secondary to volume depletion and intratubular heme pigment cast formation [1]. Lumbosacral plexopathy and compartment syndrome may develop in rare cases secondary to tissue edema [2]. The acuity, rapidity, and severity of the presenting symptoms should raise suspicion regarding the possibility of the aforementioned complications aggravating the effects of rhabdomyolysis.

The lumbosacral plexus is a network of nerves formed by the anterior rami of the lumbar and sacral spinal cord [2]. It innervates the lower back, pelvis, and legs. Lumbosacral plexopathy can result from compression from surrounding structures, masses, or edema [2]. It presents with lower back and lower extremity weakness, pain, paresthesia, numbness, or tingling [2]. When severe, it can lead to intractable pain, progressive neurological deterioration, and joint contracture [2]. Treatment often centers around promptly addressing the underlying etiology with a view to preventing further nerve damage [2].

Acute immune-mediated demyelinating polyneuropathy is a syndrome of neuronal demyelination secondary to an inciting event that triggers an immune response that cross-reacts with shared epitopes on peripheral nerves, a process called molecular mimicry [3]. Patients affected by this syndrome exhibit similar myopathic symptoms as in rhabdomyolysis, thus making it challenging to clinically distinguish between the two, especially when coupled with albumin-cytologic dissociation on CSF analysis [4]. In this case, however, MRI with contrast of the affected limbs in the acute symptomatic phase of rhabdomyolysis showed an unusual symmetrical pattern of muscle edema with symmetrical contrast enhancement (Figures 1, 2). These MRI findings coupled with the clinical picture of significantly elevated creatinine kinase, and the absence of any evidence of demyelinating process on MRI with contrast led both the internal medicine service and the neurology service to conclude acute inflammatory demyelinating polyneuropathy (AIDP) was highly unlikely. The albumin-cytologic dissociation seen on the CSF analysis was deemed to be most consistent with chronic degenerative changes.

In an adult patient with rapid, bilateral lower extremity weakness and sensory deficits, and albumin-cytologic dissociation, MRI with contrast can be coupled with creatinine kinase levels to narrow the differentials down to severe rhabdomyolysis with lumbosacral plexopathy. Narrowing the differential down in this manner allows clinicians to administer specific treatments while avoiding unnecessary interventions such as IVIG. If evidence of hypothyroidism is detected, then concurrent thyroid replacement therapy should be administered.

## Conclusions

Rhabdomyolysis is a syndrome defined as the rapid breakdown of striated skeletal muscle that can be diagnosed through clinical and laboratory testing. However, due to its vast array of possible presentations and complications that are shared with multiple other syndromes, the decision to exclusively administer treatment for it can be difficult. MRI in rhabdomyolysis shows unusual symmetrical patterns of muscle edema with contrast enhancement. These MRI findings cannot be considered exclusive to rhabdomyolysis; however, when coupled with the clinical picture, they can serve to narrow down the differential diagnoses, assess the degree of muscle damage, raise suspicion for compressive neuropathy, and subsequently allow for a more accurate, safer, and cost-effective management plan.
